# Detection of the Deep-Sea Plankton Community in Marine Ecosystem with Underwater Robotic Platform

**DOI:** 10.3390/s21206720

**Published:** 2021-10-10

**Authors:** Jiaxing Wang, Mingqiang Yang, Zhongjun Ding, Qinghe Zheng, Deqiang Wang, Kidiyo Kpalma, Jinchang Ren

**Affiliations:** 1 School of Information Science and Engineering, Shandong University, Jinan 266237, China; 17854264303@163.com (J.W.); 15005414319@163.com (Q.Z.); wdq_sdu@sdu.edu.cn (D.W.); 2 Shenzhen Research Institute, Shandong University, Shenzhen 518000, China; 3 China National Deep Sea Center, Qingdao 266237, China; 4 IETR-INSA UMR-CNRS 6164, 35708 Rennes, France; kidiyo.kpalma@insa-rennes.fr; 5 Centre for Excellence in Signal and Image Processing, Department of Electronic and Electrical Engineering, University of Strathclyde, Glasgow G1 1XQ, UK; jinchang.ren@strath.ac.uk

**Keywords:** image motion analysis, image processing, optical flow, underwater robotic

## Abstract

Variations in the quantity of plankton impact the entire marine ecosystem. It is of great significance to accurately assess the dynamic evolution of the plankton for monitoring the marine environment and global climate change. In this paper, a novel method is introduced for deep-sea plankton community detection in marine ecosystem using an underwater robotic platform. The videos were sampled at a distance of 1.5 m from the ocean floor, with a focal length of 1.5–2.5 m. The optical flow field is used to detect plankton community. We showed that for each of the moving plankton that do not overlap in space in two consecutive video frames, the time gradient of the spatial position of the plankton are opposite to each other in two consecutive optical flow fields. Further, the lateral and vertical gradients have the same value and orientation in two consecutive optical flow fields. Accordingly, moving plankton can be accurately detected under the complex dynamic background in the deep-sea environment. Experimental comparison with manual ground-truth fully validated the efficacy of the proposed methodology, which outperforms six state-of-the-art approaches.

## 1. Introduction

Plankton are organisms that live in oceans and fresh water [1] that play an important role in the material and energy recycling within the marine food chain [2]. The study of plankton community and plankton itself is indispensable for understanding of marine resources and the impacts of climate change on ecosystems [3]. In addition, the number of plankton is a key indicator of carbon and energy cycling [4], and of great significance to species diversity and ecosystem diversity [5]. From the early 19th century to date, many examples of large-scale sensor equipment were used to solve the challenge of getting reliable high-resolution estimates of plankton abundance at depth [6]. Acoustic and optical techniques for the in-situ observation of zooplankton are currently popularly used for plankton distribution assessment. Although acoustic-based observation has outstanding advantages of high observation frequency, it has inaccurate quantification and usually requires the combination of optical image analysis or other traditional sampling of zooplankton. In recent years, a series of advances were made in computer vision [7], including hyperspectral imaging [8], principal component analysis of images [9,10], and deep learning [11,12,13] for image classification [14]. As marine plankton is small and uneven in size, it is difficult to describe it quantitatively, such as with inventory and abundance statistics.

At present, a lot of plankton detection methods are proposed that often rely heavily on the use of sophisticated underwater instruments. J. Craig et al. [15,16] constructed an ICDeep system, based on the Image Intensified Charge Coupled Device (ICCD) camera, to assess the quantity of low-light bioluminescent sources in the marine environment. Philips et al. [17] created a marine biological detector, where a Scientific CMOS (SCMOS) camera was used to image the organisms before conducting statistical analysis of the plankton abundance. With the development of the computer vision, multitarget tracking-enabled automatic analysis was gradually applied to this field [18]. Kocak et al. [19] proposed to use the active contour (snake) models to segment, label, and track images of the snake model for the classification of the plankton. Luca et al. [20] also presented an automatic plankton counting method, which mainly used the interframe difference and the intersection of the bounding boxes to perform multitarget matching. The aforementioned methods achieved some results in automatic analysis and counting. However, there are still some challenges due to the particularity and complexity of plankton’s own form and passive movement mode. Applying machine vision techniques to underwater images or videos is a feasible way to study plankton at present. Underwater plankton imaging has the capacity to detect patterns of the plankton distributions that we would be unable to be tackled by sampling with nets. [21]. Therefore, we consider applying machine vision technology to underwater images or videos is currently a feasible method for studying plankton.

**Figure 1:**
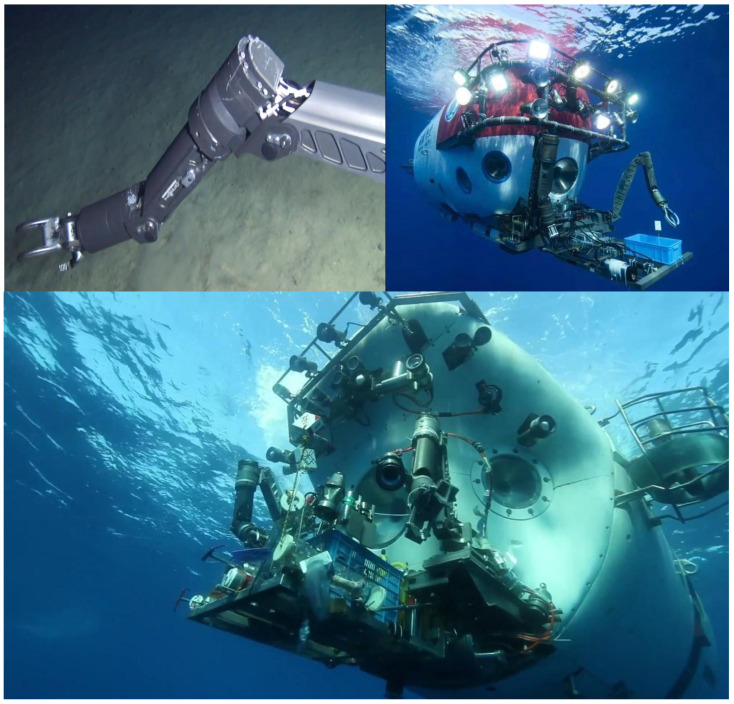
Underwater robot pattern: submarine can push and control underwater robot to complete collection of deep-sea data, then store data in computer for analysis.

Underwater robots play an important role in various video surveillance tasks including data collection. A mobile robot that can be fixed on a rotatable axis would be advantageous because it provides $360^{\circ }$ visual coverage instead of using a fixed image camera installed in a predetermined direction. These mobile robots capture unprecedented shots of marine life in dangerous environments inaccessible to humans. A submarine can push and control the underwater robot to complete the collection of deep-sea data and store the data in the computer for analysis. Some underwater robots are shown in Figure 1.

In this paper, we propose a deep-sea plankton detection method based on the Horn–Schunck (HS) optical flow [22]. The optical flow is the instantaneous velocity of the pixel movement of the moving object on the image plane. The advantage of the optical flow method is that the motion vectors can be estimated by the optical flow vector accurately. In this way, one can detect the plankton and easily analyze statistically its volume using image processing and machine vision. The research on plankton can be specifically divided into density, position, number, individual and total volume, etc. In the case where the spatial position of plankton does not coincide in two consecutive frames, the presence or absence of plankton should be determined according to the following conditions: the time gradient maps at the plankton’s location in two consecutive optical flow fields will be opposite to each other, and the horizontal and vertical gradients of the plankton at that location are equal and their direction is the same. Since the connected components are marked as the location of plankton, the number of connected components can be regarded as the number of plankton. By using this method, we firstly count the number of plankton in the video, followed by a statistical analysis. Various comparative experiments are carried out to benchmark with other methods to fully demonstrate the effectiveness of the proposed methodology.

## 2. The Proposed Method

### 2.1. Principle

**Figure 2:**
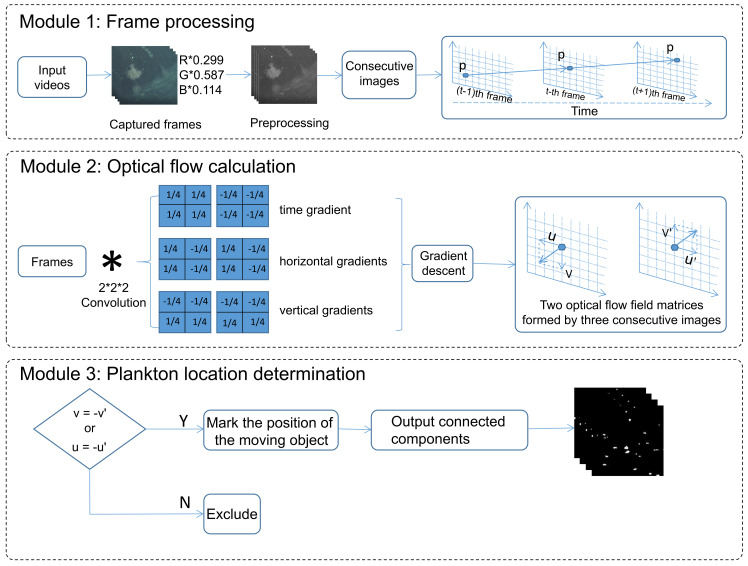
Main processing blocks of proposed algorithm. Module 1 is for preprocessing, whilst Module 2 performs 3D convolution on video frame to extract dense optical flow. Module 3 is a dual threshold setting to determine whether a plankton is contained at a specific location or not(see Section 3.3).

The deep ocean floor is clear and suitable for video acquisition with active lighting. During the video acquisition process, the camera position and shooting angle change with the movement of the submersible, making the plankton detection task a moving target detection problem under complex and dynamic backgrounds. Two consecutive optical flow field matrices derived from three consecutive video frames in a video are employed. For fast-moving plankton (plankton does not overlap in space in two consecutive frames), the two consecutive optical flow values at the position where the plankton is located are opposite. In practice, the amount of grayscale change is often close to 0. Therefore, the two consecutive optical flows are approximately opposite to each other, and we discuss this situation by setting two thresholds in the experiment section. We use this property to map out the location of the plankton. Figure 2, hereafter provides an overview of the proposed method, which consists of three modules.

**Figure 3:**
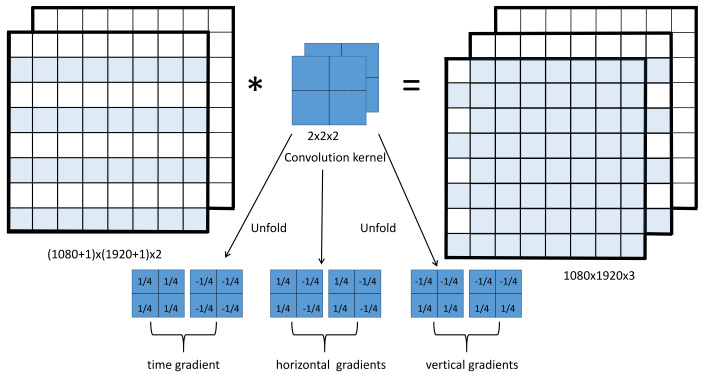
Three convolution kernels corresponding in time and space. Two consecutive frames are used to form a 3D matrix whose size is (height+1)×(width+1)×2. Size of filter is 2×2×2. Result of each operation is gradient of the pixel at upper-left corner of convolution kernel.

As shown in the Module 1 of Figure 2, grayscale images are obtained by weighting three channels of the input frames. In module 2, three convolution operations are performed on two consecutive frames to produce three different gradients(see Figure 3), which correspond to three different convolution kernels. The details of the convolution process are shown in Figure 3 to illustrate this process. We find that the time gradients of the two optical flow fields derived from three consecutive frames of images are opposite in numerical value and direction in the corresponding positions of plankton in the middle frame. In the following description, the time gradients of the two consecutive optical flow fields are represented by $\nabla_{t}$ and $\nabla_{t}^{\prime }$. The horizontal gradients of the two consecutive optical flow fields derived from three consecutive frames are equal in magnitude and direction in the corresponding positions of plankton in the middle frame. Similarly, the vertical gradients are also equal. In the following description, the horizontal gradients of the two optical flow fields are represented by $\nabla_{x}$ and $\nabla_{x}^{\prime }$, the vertical gradients are $\nabla_{y}$ and $\nabla_{y}^{\prime }$. Finally, Module 3 is for dual thresholding, which is explained separately when discussing the parameters later.

### 2.2. Proof

**Figure 4:**
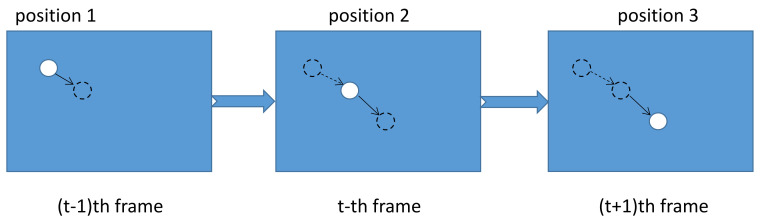
Position of plankton in three consecutive frames.

In the HS optical flow method, the constraint equation of optical flow can be established as Equation (2) according to the premise of the optical flow method: invariance of gray level [22]. Three first-order differences are used to replace the horizontal, vertical, and time gradients. Let the gray value at plankton’s position in the middle frame be $I_{x,y,t}$, where the subscripts *x* and *y* are the pixel index, and *t* is the time index. The position of plankton changes with the movement of ocean current and the camera lens. As shown in Figure 4, the plankton is small-sized, so its position in frame *t* doesn’t overlap in frame *t* + 1. When it changes from position 1 to position 2, the gray value corresponding to position 2 of plankton at frame *t* − 1 is the background gray value $I_{x,y,t-1}$. In a similar way, when the position of plankton changes from position 2 to position 3, the gray value corresponding to position 2 at frame *t* + 1 becomes the background gray value $I_{x,y,t+1}$. Based on the characteristics of deep-sea underwater video, the background around the plankton is invariant in time, i.e.,:

(1) $$I_{x,y,t-1}=I_{x,y,t+1}$$

(2) $$\nabla_{x}u+\nabla_{y}v+\nabla_{t}=0$$

The time gradients at the plankton’s positions in the two adjacent optical flow fields are:

(3) $$\nabla_{t}=\frac{1}{2}\left(I_{x,y,t}-I_{x,y,t-1}+I_{x+1,y,t}-I_{x+1,y,t-1}\right)$$

(4) $$\nabla_{t}^{\prime }=\frac{1}{2}\left(I_{x,y,t+1}-I_{x,y,t}+I_{x+1,y,t+1}-I_{x+1,y,t}\right)$$

Based on Equation (1), the background gray value $I_{x,y,t-1}=I_{x,y,t+1}$, $\nabla_{t}=-\nabla_{t}^{\prime }$, the time gradients of the two optical flow fields derived from three consecutive frames of images are opposite in the corresponding positions of plankton in the middle frame.

The horizontal gradients of the plankton’s location in the two optical flow fields are:

(5) $$\nabla_{x}=\frac{1}{2}\left(I_{x+1,y,t}-I_{x,y,t}+I_{x+1,y,t-1}-I_{x,y,t-1}\right)$$

(6) $$\nabla_{x}^{\prime }=\frac{1}{2}\left(I_{x+1,y,t+1}-I_{x,y,t+1}+I_{x+1,y,t}-I_{x,y,t}\right)$$

The same way, based on Equation (1), we can get that $\nabla_{x}=\nabla_{x}^{\prime }$, i.e., the horizontal gradients of the two optical flow fields derived from three consecutive frames are equal in the corresponding positions of plankton in the middle frame. In the same way, we can get $\nabla_{y}=\nabla_{y}^{\prime }$.

In fact, in the process of proof, the time and space gradients are estimated in a 2×2×2 cubic neighborhood by taking the mean.

Then, we iterate *n* times for gray gradient relaxation by setting the initial conditions as $v_{0}=v_{0}^{\prime }=0$ and $u_{0}=u_{0}^{\prime }=0$.

(7) $$\Delta =(\frac{\nabla_{x}u_{n}+\nabla_{y}v_{n}+\nabla_{t}}{\alpha_{2}+\nabla_{x}^{2}+\nabla_{y}^{2}})$$

(8) $$u_{n+1}=u_{n}-\nabla_{x}\Delta$$

(9) $$v_{n+1}=v_{n}-\nabla_{y}\Delta$$

The parameter $\alpha_{2}$ reflects the smoothness constraints of the HS optical flow algorithm; Δ is an iteration factor in the process of the iterative algorithm; $\nabla_{x}$ and $\nabla_{y}$ are the horizontal and vertical gradients, and *u* and *v* are the horizontal and vertical optical flow field matrices, respectively.

The relationships of Equations (7)–(9) are represented by a series, where the number of iterations is *n*. Let’s substitute Equations (7)–(9), the new formulas are as follows:

(10) $$u_{n+1}=u_{n}-\nabla_{x}\left(\frac{\nabla_{x}u_{n}+\nabla_{y}v_{n}+\nabla_{t}}{\alpha_{2}+\nabla_{x}^{2}+\nabla_{y}^{2}}\right)$$

(11) $$v_{n+1}=v_{n}-\nabla_{y}\left(\frac{\nabla_{x}u_{n}+\nabla_{y}v_{n}+\nabla_{t}}{\alpha_{2}+\nabla_{x}^{2}+\nabla_{y}^{2}}\right)$$

where $u_{n+1}$ and $u_{n}$ are two horizontal optical flow fields before and after the *n*-th iteration, $v_{n+1}$ and $v_{n}$ are two vertical optical flow fields before and after the *n*-th iteration. We can derive $u_{n+1}=-u{}_{n+1}^{\prime }$, $v_{n+1}=-v{}_{n+1}^{\prime }$. When *n* = 0, we have:

(12) $$v_{1}=v_{0}-\nabla_{y}\left(\frac{\nabla_{x}u_{0}+\nabla_{y}v_{0}+\nabla_{t}}{\alpha_{2}+\nabla_{x}^{2}+\nabla_{y}^{2}}\right)$$

(13) $$v_{1}^{\prime }=v_{0}^{\prime }-\nabla_{y}^{\prime }\left(\frac{\nabla_{x}^{\prime }u_{0}^{\prime }+\nabla_{y}^{\prime }v_{0}^{\prime }+\nabla_{t}^{\prime }}{\alpha_{2}+\nabla_{x}^{{}^{\prime }2}+\nabla_{y}^{{}^{\prime }2}}\right)$$

$v_{1}$ and $v_{1}^{\prime }$ are the two consecutive vertical optical flow field at the first iteration. If the time gradients of the last two optical flow fields are opposite, that is $\nabla_{t}=-\nabla_{t}^{\prime }$, we can get: $v_{1}=-v{}_{1}^{\prime }$.

When n=k, $v_{k+1}=-v{}_{k+1}^{\prime }$. That is, Equations (14) and (15) are opposite:

(14) $$v_{k+1}=v_{k}-\nabla_{y}\left(\frac{\nabla_{x}u_{k}+\nabla_{y}v_{k}+\nabla_{t}}{\alpha_{2}+\nabla_{x}^{2}+\nabla_{y}^{2}}\right)$$

(15) $$v{}_{k+1}^{\prime }=v{}_{k}^{\prime }-\nabla_{y}^{\prime }\left(\frac{\nabla_{x}u{}_{k}^{\prime }+\nabla_{y}^{\prime }v{}_{k}^{\prime }+\nabla_{t}^{\prime }}{\alpha_{2}+\nabla_{x}^{{}^{\prime }2}+\nabla_{y}^{{}^{\prime }2}}\right)$$

where $v_{k+1}$ and $v{}_{k+1}^{\prime }$ represent the previous and the next vertical optical flow field matrix at the (*k* + 1)th iteration, respectively.

When n=k+1, we can show that $v_{k+2}=-v{}_{k+2}^{\prime }$

(16) $$v_{k+2}=v_{k+1}-\nabla_{y}\left(\frac{\nabla_{x}u_{k+1}+\nabla_{y}v_{k+1}+\nabla_{t}}{\alpha_{2}+\nabla_{x}^{2}+\nabla_{y}^{2}}\right)$$

(17) $$v{}_{k+2}^{\prime }=v{}_{k+1}^{\prime }-\nabla_{y}^{\prime }\left(\frac{\nabla_{x}^{\prime }u{}_{k+1}^{\prime }+\nabla_{y}^{\prime }v{}_{k+1}^{\prime }+\nabla_{t}^{\prime }}{\alpha_{2}+\nabla_{x}^{{}^{\prime }2}+\nabla_{y}^{{}^{\prime }2}}\right)$$

By adding Equations (16) and (17), and substituting $v_{k+1}=-v{}_{k+1}^{\prime }$, $\nabla_{x}=\nabla_{x}^{\prime }$ and $\nabla_{y}=\nabla_{y}^{\prime }$ into Equation (16) and Equation (17), respectively, we have:

(18) $$v_{k+2}=-v{}_{k+2}^{\prime }$$

Therefore, for fast-moving plankton, the values of the vertical optical flow field matrices of the space position where the plankton is located are opposite from each other: $v=-v{}^{\prime }$, and the same applies horizontally: $u=-u{}^{\prime }$.

### 2.3. The Volume of Plankton

Based on the above proof, one can calculate the number of pixels where plankton is located, and then multiply the actual size of a pixel to obtain the area of plankton. The resolution of the known image is height×width. According to camera internal reference, the actual range of our field of view is about W m by H m. The calculation of the actual area is given by:

(19) S=N×(W/width)×(H/height)

where *N* is the number of pixels, and *S* is the corresponding actual surface. A method of approximate calculation is adopted here. Firstly, we can get the radius of a circle that has the same area as the plankton, and then calculate the volume of the sphere based on that radius. The advantage of this method is that we can get the 3D volume of an irregular object only by its area [23]. In addition, we can predict the type of plankton based on the estimated size, laying the foundation for the later identification of plankton types. The volume can be calculated by:

(20) $$V=\frac{4}{3}\times \pi^{-\frac{1}{2}}\times S^{\frac{3}{2}}$$

The proposed method adds its own theoretical innovation on the basis of the original optical flow method and was proved mathematically. In this way, the complexity and passive motion patterns of plankton are well-solved, and the accuracy improves as the above problems are solved.

## 3. Experimental Results and Analysis

**Figure 5:**
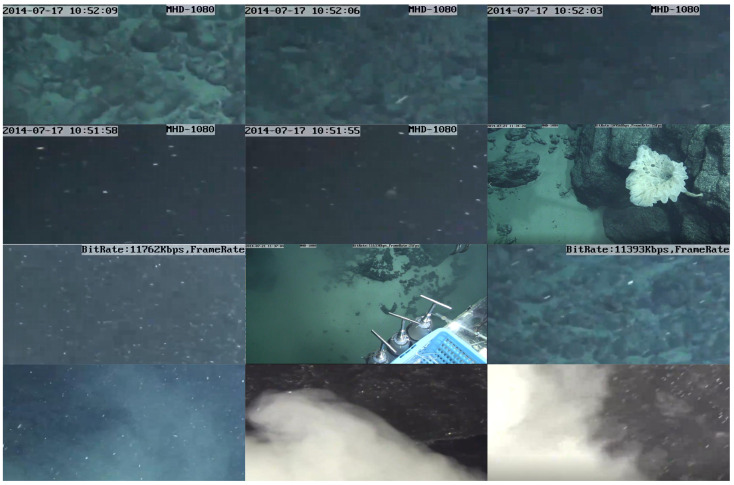
Example images of deep sea plankton.

The data capture was provided by the China National Deep Sea Center. The data set was obtained by an underwater robotic nondestructive testing system carried by a deep-sea manned submersible. The camera’s technical specifications are: resolution: 1080*i* HDTV; minimum illumination: 2l ux; optical zoom: 10 times; digital zoom: 12 times; aperture range: 3.2 mm–32 mm; video aspect ratio: 16:9 or 4:3. In this study, three six-minute videos of the plankton community from appearing to disappearing from the screen were selected, which were obtained from a submarine on the western Pacific sea mountain slope, and the diving depths are 2741.88 m and 5555.68 m, corresponding to 76 and 77 dives, respectively. The reason why the three videos are selected is that plankton appeared more frequently in them. Due to the complexity of the deep-sea environment and the irregular camera movement, the background is complex and dynamic. In this case, using high-precision image processing technology to study the plankton community from appearing to disappearing from the screen can effectively distinguish sedimentary clouds and plankton community in images. Examples of deep-sea plankton images are shown in Figure 5 and the details of data set including diving number, date, diving time, longitude, latitude and depth are shown in Table 1.

### 3.1. Number and Volume of Plankton

**Figure 6:**
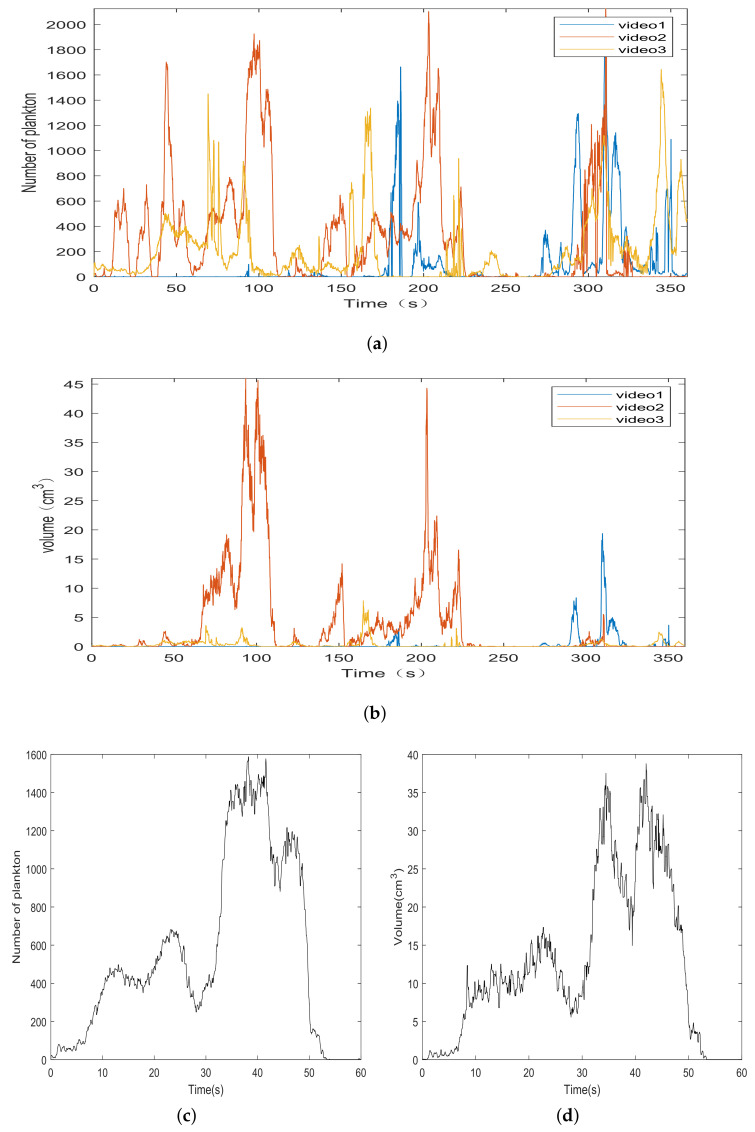
(**a**) Number of plankton in three six-minute videos. (**b**) Total volume of plankton in three six-minute videos. (**c**) Number of plankton in a period. (**d**) Volume of plankton in a period.

Processing the recorded video of a complete plankton community from appearing to disappearing from the screen, the results obtained are shown in Figure 6. Figure 6a shows the variation of the number of plankton in three six-minute videos, and Figure 6b shows the variation of the volume of the corresponding three videos. The process of plankton appearing in front of the camera to disappearing is shown in Figure 6c,d. In the first 30 s of Figure 6c, the amount of plankton is small and the detection results are more accurate. We can see that the amount of plankton rises in the last 30 s of Figure 6c. For dense particle clouds, overlap, and hence, occlusion occurs frequently, which leads to relatively low average accuracy and recall rates.

The actual volume curve of plankton in the video is shown in Figure 6b,d. We can see that the volume curve and the quantity curve of plankton generally follow the same trend. At the 40th second in Figure 6c, the plankton community moves away from the camera and then comes back, resulting in a smaller scene and a smaller overall volume due to perspective. So, we can see that the volume curve goes down and then goes up from Figure 6d.

### 3.2. Comparison with Six Target Detection Methods

**Figure 7:**
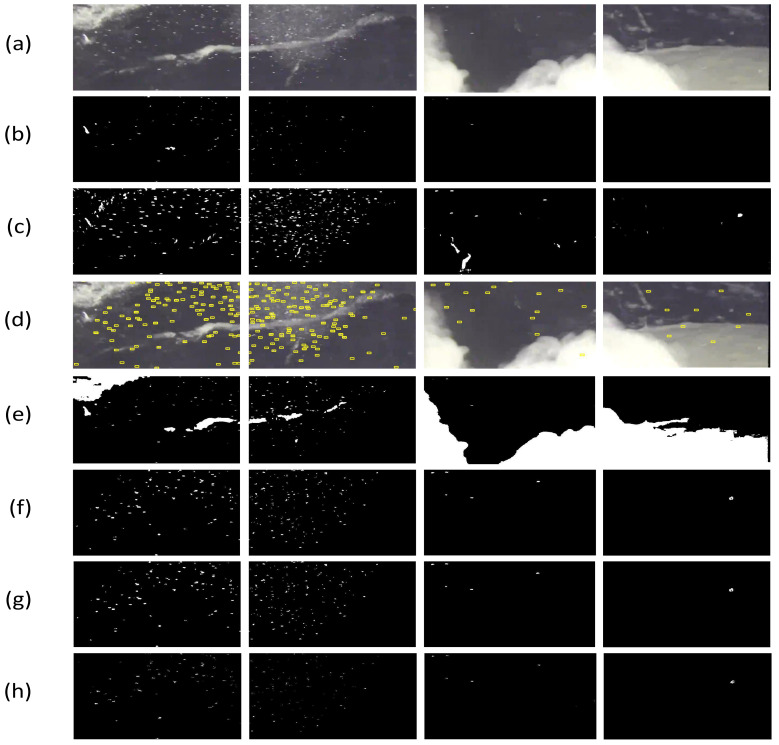
Location of plankton detected with seven different methods: (**a**) original image; (**b**) Top-Hat transform; (**c**) frame difference method; (**d**) motion estimation and image match; (**e**) scan line marking method; (**f**) simple block-based sum of absolute differences flow (SD); (**g**) Lucas–Kanade (LK) optical flow method, and (**h**) proposed method.

The proposed method is compared with six state-of-the-art methods for performance evaluation. The results are shown in Figure 7, where Figure 7a represents some original images of the video, including sediment clouds, plankton, and uneven backgrounds. Top-Hat transform [24] is used to detect the location of the plankton in the image as shown in Figure 7b, the weakness of this algorithm is that there are some missed cases. Figure 7c and Figure 7d show the detection results of the frame difference method [25] and the motion estimation and image matching method [26], respectively. We show the result from the scan line marking method [27] in Figure 7e results from the simple block-based sum of absolute differences flow (SD) method [28], and the Lucas–Kanade (LK) optical flow method [29] are given in Figure 7f,g. The weakness of the above three methods is that there are a few false positives, and both Figure 7c,e detected the sediment cloud in the background by mistake. The result of Figure 7h is obtained using the proposed method. After comparing with the manual ground truth, we find that the plankton detected by the proposed method is more consistent with the original image in Figure 7a.

We take 20 images of the video, and the data are cleaned by manual counting to get the ground-truth. Then, we compare the number of plankton, recall rate, precision rate, and F1-score of the seven methods. When using 10 frames in the first 30 s of the video, the amount of plankton is small and the detection results are more accurate, the average accuracy rate is 0.901, the average recall rate is 0.955, and F1-score is 0.927. In addition, the equations and related symbols are shown in Table 2 and Equations (21)–(23). The results are shown in Table 3 and Table 4. Taking 10 frames in the last 30 s of the video, the amount of plankton is high. For dense particle clouds, overlap can easily occur, and hence, occlusion occurs frequently, so the average accuracy and recall rates are relatively low, i.e., 0.895 and 0.943, respectively, and the F1-score is 0.918, The results are shown in Table 5 and Table 6. In addition, we randomly selected 10 frames from the video for testing. The experimental results are shown in Table 7 and Table 8. The performance of the proposed method is still very good. We use bold font to highlight the best results in each category in Table 4, Table 6, Table 8 and Table 9.

(21) $$Precision=\frac{TP}{TP+FP}$$

(22) $$Recall=\frac{TP}{TP+FN}$$

(23) $$F1=\frac{2Precision\times Recall}{Precision+Recall}$$

### 3.3. Discussion of Parameters

For each imaging system, there is a depth of field within which the closest field objects and farthest field objects are all in focus. If we deploy the system in air, the light intensity for the near field object and far field object should not be different in theory. However, when deployed in seawater, the light intensity changes as the light propagates in the water from near-field to far-field because of scattering caused by seawater and particles in the seawater. Therefore, during the experiment, there are two situations that need to be discussed. Firstly, ’grayscale invariance’ is one of the prerequisites of the HS optical flow method, but in actual operation, the amount of grayscale change is often close to 0 but not equal to 0. Therefore, the threshold $\beta_{1}$ is set to handle this situation, as shown in Equation (24).

(24) $$\left|u+u{}^{\prime }\right|<\beta_{1}\;\;or\;\left|v+v{}^{\prime }\right|<\beta_{1}\;$$

Secondly, when there is no plankton and the optical flow happens to be small, if the values of the optical flow are not the opposite but the sum still conforms to Equation (24), the threshold $\beta_{2}$ needs to be set to solve this situation, as shown in Equation (25).

(25) $$-uu{}^{\prime }>\beta_{2}\;\;or\;-vv{}^{\prime }>\beta_{2}\;$$

The best threshold value is obtained by traversing the range value, the scope of $\beta_{1}$ is 0.05 to 0.35, step size is 0.05, the scope of $\beta_{2}$ is 3–9, and the step length is 1. Then, the original images and all those resulting from different thresholds are represented by vectors. At last, we calculate the cosine similarity between two images, that is the calculation of cosine distance between two vectors; the larger the cosine distance between the two vectors, the more similar the two images are. The results are shown in Table 9.

**Figure 8:**
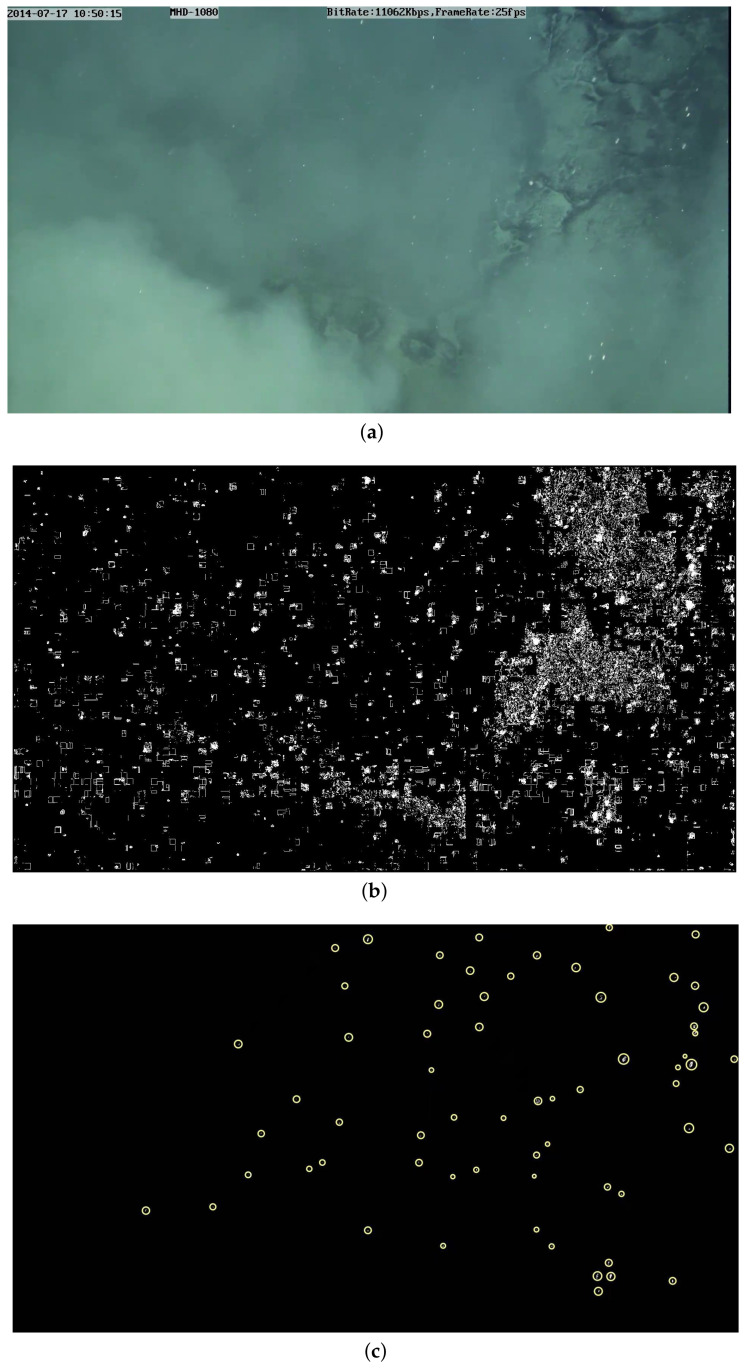
Comparison with or without threshold: (**a**) original image; (**b**) one without threshold $\beta_{2}$, and (**c**) result of using threshold $\beta_{2}$.

As shown in Figure 8, Figure 8a is the original image, Figure 8c represents the result of using the threshold $\beta_{2}$, and the one without the threshold $\beta_{2}$ is shown in Figure 8b.

### 3.4. Time Complexity Comparison

The time complexity comparison of the proposed method and six state-of-the-art methods is provided in Table 10. We select a one-minute video of 1440 frames and calculate the computation time to measure the time complexity of difference methods. Although the proposed method doesn’t have great advantage in term of the time complexity, it outperforms other methods in accurate detection of plankton. In terms of the detection efficiency, some experimental comparisons were carried out. Based on the same one-minute video, the computation time and recall rate of the following four different strategies are compared, respectively. We sample pixels at intervals of 1, and take interval frames from full sequence at intervals of 1 frame. According to the results shown in Table 11, the interval between pixels has a weak influence on the error of the result, where the recall rate, precision rate, and F1-score are the closest to the original image’s result, and the detection efficiency is improved by greatly reducing the calculation time.

## 4. Conclusions

Detection of plankton plays an important role in the exploration and research of deep-sea areas. Variations in the quantity and spatial distribution of plankton determine the function of the entire marine ecosystem. In this paper, we introduce a method for deep-sea plankton community detection in marine ecosystem with an underwater robotic platform. Compared with that of traditional methods, our method simultaneously improves the precision and recall of plankton detection. The obtained results and the proved theory provide a scientific basis for studying the material cycle and energy flow of deep-sea ecosystems. For our future work, with a view to strengthening the proposed solution, we aim to improve our plankton detection approach, and then conduct studies for plankton recognition and identification of their species.

## Figures and Tables

**Table 1:** Details of datasets including diving number, date, diving time, longitude, latitude, and depth.

Diving Number	Date	Diving Time	Longitude	Latitude	Depth
76	17 July 2014	8.95 h	155.32${}^{\circ }$ E–155.34${}^{\circ }$ E	15.50${}^{\circ }$ N–15.52${}^{\circ }$ N	2741.88 m
77	21 July 2014	10.33 h	154.58${}^{\circ }$ E–154.59${}^{\circ }$ E	15.70${}^{\circ }$ N–15.72${}^{\circ }$ N	5555.68 m

**Table 2:** Confusion Matrix.

	Relevant	Nonrelevant
Retrieved	True Positives (TP)	False Positives (FP)
Not Retrieved	False Negatives (FN)	True Negatives (TN)

**Table 3:** In first 30 s, comparison of number of detected plankton using seven methods and Ground-Truth.

The Ten Frames:	1	2	3	4	5	6	7	8	9	10	Mean	std
Top-Hat	10	12	9	15	17	18	18	22	24	22	16.7	4.9
Frame difference	20	22	18	22	21	17	19	15	17	14	18.5	2.7
Image match	26	24	23	21	24	17	15	15	16	16	19.7	4.1
Scan line marking	11	7	8	9	10	9	10	9	9	8	9.0	1.1
SD	17	13	11	11	13	13	12	10	12	10	12.2	1.9
LK	17	14	13	13	13	13	12	11	11	10	12.7	1.8
Proposed method	16	14	12	12	12	11	12	10	11	10	12.0	1.7
Ground-Truth	14	13	12	12	11	12	11	9	10	9	11.3	1.6

**Table 4:** In first 30 s, comparison of recall rate, precision rate, and F1-score of seven methods.

	The Ten Frames:	1	2	3	4	5	6	7	8	9	10	Average
Top-Hat	Precision	0.9	0.85	0.89	0.73	0.59	0.61	0.56	0.36	0.42	0.41	0.632
	Recall	0.64	0.92	0.67	0.92	0.91	0.92	0.91	0.89	1	1	0.878
	F1	0.75	0.88	0.76	0.81	0.72	0.73	0.69	0.51	0.59	0.58	0.73
Frame difference	Precision	0.65	0.55	0.61	0.5	0.52	0.65	0.56	0.53	0.59	0.57	0.573
	Recall	0.93	0.92	0.92	0.92	1	0.92	1	0.89	1	0.89	0.939
	F1	0.77	0.69	0.73	0.65	0.68	0.76	0.72	0.66	0.74	0.69	0.712
Image match	Precision	0.54	0.54	0.49	0.52	0.46	0.65	0.67	0.53	0.56	0.56	0.552
	Recall	1	1	0.92	0.92	1	0.92	0.91	0.89	0.9	1	0.946
	F1	0.7	0.7	0.64	0.66	0.63	0.76	0.77	0.66	0.69	0.72	0.7
Scan line marking	Precision	0.82	0.86	0.88	0.78	0.9	0.89	0.9	0.89	0.89	0.88	0.869
	Recall	0.64	0.46	58	0.58	0.82	0.67	0.82	0.89	0.8	0.78	0.704
	F1	0.72	0.6	0.7	0.67	0.86	0.76	0.86	**0.89**	0.84	0.83	0.778
SD	Precision	0.76	0.92	0.91	0.91	0.77	0.85	0.83	0.8	0.75	0.8	0.83
	Recall	0.93	0.92	0.83	0.83	0.91	0.92	0.91	0.89	0.9	0.89	0.893
	F1	0.84	0.92	0.87	0.87	0.83	0.88	**0.87**	0.84	0.82	0.84	0.86
LK	Precision	0.76	0.86	0.85	0.85	0.85	0.85	0.83	0.73	0.82	0.8	0.82
	Recall	0.93	0.93	0.92	0.92	1	0.92	0.91	0.89	0.9	0.89	0.921
	F1	0.84	0.89	0.88	0.88	0.92	0.88	**0.87**	0.8	**0.86**	0.84	0.868
Proposed method	Precision	0.81	0.93	1	1	0.92	1	0.83	0.8	0.82	0.9	0.901
	Recall	0.93	1	1	1	1	0.92	0.91	0.89	0.9	1	0.955
	F1	**0.87**	**0.96**	**1**	**1**	**0.96**	**0.96**	**0.87**	0.84	**0.86**	**0.95**	**0.927**

**Table 5:** In last 30 s, comparison of number of detected plankton using seven methods and Ground-Truth.

The Ten Frames:	1	2	3	4	5	6	7	8	9	10	Mean	std
Top-Hat	22	17	17	16	13	12	13	12	9	18	14.9	3.6
Frame difference	28	28	30	24	30	15	28	28	27	24	26.2	4.2
Image match	22	22	25	26	31	27	32	28	31	24	26.8	3.5
Scan line marking	13	15	14	11	16	15	19	15	15	13	14.6	2.0
SD	16	21	22	23	23	23	22	23	20	17	21.0	2.4
LK	16	22	23	22	23	23	21	23	20	18	21.1	2.4
Proposed method	15	21	22	21	22	21	21	22	19	16	20.0	2.4
Ground-Truth	19	19	19	18	21	18	21	21	18	15	18.9	1.8

**Table 6:** In last 30 s, comparison of recall rate, precision rate, and F1-score of seven methods.

	The Ten Frames:	1	2	3	4	5	6	7	8	9	10	Average
Top-Hat	Precision	0.77	0.94	0.94	0.93	0.92	0.92	0.92	0.92	1	0.77	0.903
	Recall	0.89	0.84	0.84	0.83	0.57	0.61	0.57	0.52	0.5	0.93	0.762
	F1	0.83	0.89	**0.89**	0.88	0.7	0.73	0.7	0.66	0.67	0.84	0.827
Frame difference	Precision	0.64	0.64	0.6	0.71	0.65	0.93	0.71	0.71	0.59	0.58	0.676
	Recall	0.95	0.95	0.95	0.94	0.95	0.78	0.95	0.95	0.88	0.93	0.923
	F1	0.76	0.76	0.74	0.81	0.77	0.85	0.81	0.81	0.71	0.71	0.78
Image match	Precision	0.82	0.82	0.72	0.65	0.67	0.63	0.63	0.71	0.55	0.58	0.678
	Recall	0.95	0.95	0.95	0.94	0.95	0.94	0.95	0.95	0.94	0.93	0.945
	F1	**0.88**	0.88	0.82	0.77	0.79	0.75	0.76	0.81	0.69	0.71	0.789
Scan line marking	Precision	0.92	0.93	0.93	1	0.94	0.93	0.95	0.93	0.93	0.92	0.938
	Recall	0.63	0.74	0.68	0.61	0.71	0.78	0.86	0.67	0.78	0.8	0.726
	F1	0.75	0.82	0.79	0.76	0.81	0.85	0.9	0.78	0.85	0.86	0.82
SD	Precision	0.94	0.85	0.82	0.74	0.87	0.74	0.91	0.87	0.85	0.82	0.841
	Recall	0.79	0.94	0.94	0.94	0.95	0.94	0.95	0.95	0.94	0.93	0.893
	F1	0.86	0.89	0.88	0.83	0.91	0.83	0.93	0.91	0.89	0.87	0.88
LK	Precision	0.94	0.82	0.78	0.77	0.87	0.74	0.95	0.87	0.85	0.78	0.837
	Recall	0.79	0.94	0.95	0.94	0.95	0.94	0.95	0.95	0.94	0.93	0.928
	F1	0.86	0.88	0.86	0.85	0.91	0.83	**0.95**	0.91	0.89	0.85	0.88
Proposed method	Precision	1	0.85	0.82	0.86	0.91	0.81	0.9	0.91	0.95	0.94	0.895
	Recall	0.79	0.95	0.95	1	0.95	0.94	0.9	0.95	1	1	0.943
	F1	**0.88**	**0.9**	0.88	**0.92**	**0.93**	**0.87**	0.9	**0.93**	**0.97**	**0.97**	**0.918**

**Table 7:** Comparison of number of detected plankton from 10 randomly selected frames.

The Ten Frames:	1	2	3	4	5	6	7	8	9	10
Top-Hat	19	34	1	47	45	3	3	0	0	2
Frame difference	281	260	10	159	143	15	13	8	10	12
Image match	78	129	12	106	120	16	15	11	9	14
Scan line marking	8	119	1	51	99	7	4	2	2	3
SD	172	195	0	83	124	1	5	1	1	9
LK	163	190	0	86	121	1	5	1	1	10
Proposed method	94	105	0	71	89	1	7	1	1	5
Ground-Truth	87	94	1	66	76	2	6	1	1	5

**Table 8:** Comparison of recall, precision, and F1-score of detected plankton from 10 randomly selected frames.

	The Ten Frames:	1	2	3	4	5	6	7	8	9	10	Average
Top-Hat	Precision	0.95	0.88	1.00	0.94	0.89	0.67	1.00	0.00	0.00	1.00	0.733
	Recall	0.21	0.32	1.00	0.66	0.53	1.00	0.50	0.00	0.00	0.40	0.462
	F1	0.34	0.47	**1.00**	0.78	0.66	**0.80**	0.67	0.00	0.00	0.57	0.529
Frame difference	Precision	0.28	0.35	0.1	0.38	0.49	0.13	0.38	0.13	0.10	0.33	0.267
	Recall	0.92	0.96	1.00	0.91	0.92	1.00	0.83	1.00	1.00	0.80	0.934
	F1	0.43	0.51	0.18	0.54	0.64	0.23	0.52	0.23	0.18	0.47	0.393
Image match	Precision	0.90	0.70	0.08	0.57	0.58	0.13	0.33	0.09	0.11	0.29	0.378
	Recall	0.80	0.96	1.00	0.91	0.92	1.00	0.83	1.00	1.00	0.80	0.922
	F1	0.85	0.81	0.15	0.70	0.71	0.23	0.47	0.17	0.20	0.43	0.472
Scan line marking	Precision	0.94	0.73	**1.00**	0.88	0.71	0.29	1.00	0.50	0.50	0.67	0.722
	Recall	0.86	0.93	1.00	0.68	0.92	1.00	0.67	1.00	1.00	0.40	0.846
	F1	0.90	0.82	1.00	0.77	0.80	0.45	0.80	0.67	0.67	0.50	0.738
SD	Precision	0.47	0.46	0.00	0.72	0.57	1.00	1.00	1.00	1.00	0.56	0.678
	Recall	0.93	0.95	0.00	0.91	0.93	0.50	0.83	1.00	1.00	1.00	0.805
	F1	0.62	0.62	0.00	0.80	0.71	0.67	0.91	**1.00**	**1.00**	0.72	0.705
LK	Precision	0.50	0.47	0.00	0.72	0.57	1.00	1.00	1.00	1.00	0.50	0.676
	Recall	0.94	0.95	0.00	0.94	0.91	0.50	0.83	1.00	1.00	1.00	0.807
	F1	0.65	0.63	0.00	0.82	0.70	0.67	0.91	**1.00**	**1.00**	0.67	0.705
Proposed method	Precision	0.88	0.86	0.00	0.89	0.81	1.00	0.86	1.00	1.00	1.00	0.830
	Recall	0.95	0.96	0.00	0.95	0.95	0.50	1.00	1.00	1.00	1.00	0.831
	F1	**0.91**	**0.91**	0.00	**0.92**	**0.87**	0.67	**0.92**	**1.00**	**1.00**	**1.00**	**0.820**

**Table 9:** Select best threshold by comparing cosine distance between two vectors.

Threshold:	0.05	0.10	0.15	0.20	0.25	0.30	0.35
3	0.050	0.059	0.065	0.069	0.072	0.074	**0.076**
4	0.048	0.057	0.062	0.066	0.069	0.072	0.074
5	0.047	0.055	0.061	0.065	0.068	0.070	0.072
6	0.046	0.054	0.059	**0.076**	0.069	0.066	0.070
7	0.045	0.053	0.058	0.062	0.065	0.067	0.069
8	0.044	0.052	0.057	0.061	0.064	0.066	0.068
9	0.044	0.051	0.056	0.060	0.063	0.065	0.067

**Table 10:** Time complexity comparison of proposed method and 6 state-of-the-art methods in a 1-min video of 1440 frames.

Top-Hat	Frame Difference	Image Match	Scan Line Marking	SD	LK	Proposed Method
3478 s	176 s	13,149 s	4476 s	989 s	1070 s	1112 s

**Table 11:** Time complexity comparison of different sampling in a 1-min video of 1440 frames.

A Total of 116 Plankton	Take Interval Frames from full Sequence				
	Quantity	Precision	Recall	F1	Calculation Time
Pixel interval sampling	81	0.86	0.6	0.71	137 s
All the pixels	30	0.83	0.23	0.36	618 s
**A Total of 116 Plankton**	**Full Sequence**				
	**Quantity**	**Precision**	**Recall**	F1	**Calculation Time**
Pixel interval sampling	110	0.95	0.91	0.93	436 s
Full sequence	113	0.97	0.95	0.96	1112 s

## Data Availability

Not applicable.
